# Upon Fluids Which Stop Hæmorrhage

**Published:** 1853-11

**Authors:** 


### SURGERY.

*Upon Fluids which stop Hæmorrhage.* By SEDILLOT.

*Eau de Pagliari*, consisting of Tinct. Benzoes, ℥viii.
Alum, lb. i.
Water, lb. x.

This preparation, which has a pale straw color, and is transparent, possesses an extraordinary power of coagulating blood.

*Eau de Rabel*, consisting of Acid. sulphur., 100 parts.
Alcohol, 300 parts.

The acid should be poured upon the alcohol slowly.

*Baume de Compignt* has not yet been analysed, but appears to contain an empyreumatic oil.

*Eau de Hepp* is a modification of the Eau de Pagliari.—*London Med. Times.*

